# Genome-Wide Association Study Uncovers Novel Genomic Regions Associated With Coleoptile Length in Hard Winter Wheat

**DOI:** 10.3389/fgene.2019.01345

**Published:** 2020-02-05

**Authors:** Jagdeep Singh Sidhu, Dilkaran Singh, Harsimardeep Singh Gill, Navreet Kaur Brar, Yeyan Qiu, Jyotirmoy Halder, Rami Al Tameemi, Brent Turnipseed, Sunish Kumar Sehgal

**Affiliations:** ^1^ Department of Agronomy, Horticulture & Plant Science, South Dakota State University, Brookings, SD, United States; ^2^ Department of Biology and Microbiology, South Dakota State University, Brookings, SD, United States

**Keywords:** *Triticum aestivum*, coleoptile length, semi-dwarf wheat, genome-wide association study, quantitative trait loci, SNP (Single-nucleotide polymorphism), marker-assisted selection

## Abstract

Successful seedling establishment depends on the optimum depth of seed placement especially in drought-prone conditions, providing an opportunity to exploit subsoil water and increase winter survival in winter wheat. Coleoptile length is a key determinant for the appropriate depth at which seed can be sown. Thus, understanding the genetic basis of coleoptile length is necessary and important for wheat breeding. We conducted a genome-wide association study (GWAS) using a diverse panel of 298 winter wheat genotypes to dissect the genetic architecture of coleoptile length. We identified nine genomic regions associated with the coleoptile length on seven different chromosomes. Of the nine genomic regions, five have been previously reported in various studies, including one mapped to previously known *Rht-B1* region. Three novel quantitative trait loci (QTLs), *QCL.sdsu-2AS*, *QCL.sdsu-4BL*, and *QCL.sdsu-5BL* were identified in our study. *QCL.sdsu-5BL* has a large substitution effect which is comparable to *Rht-B1*'s effect and could be used to compensate for the negative effect of *Rht-B1* on coleoptile length. In total, the nine QTLs explained 59% of the total phenotypic variation. Cultivars ‘Agate’ and ‘MT06103’ have the longest coleoptile length and interestingly, have favorable alleles at nine and eight coleoptile loci, respectively. These lines could be a valuable germplasm for longer coleoptile breeding. Gene annotations in the candidate regions revealed several putative proteins of specific interest including cytochrome P450-like, expansins, and phytochrome A. The QTLs for coleoptile length linked to single-nucleotide polymorphism (SNP) markers reported in this study could be employed in marker-assisted breeding for longer coleoptile in wheat. Thus, our study provides valuable insights into the genetic and molecular regulation of the coleoptile length in winter wheat.

## Introduction

Successful crop stand establishment is the first critical step for achieving a high yield potential (Rebetzke et al., 2007b; Rebetzke et al., 2014). Temperature and moisture are two major environmental factors that determine the success of seedling emergence out of the soil (Jame and Cutforth, 2004; Hunt et al., 2018). Therefore, to ensure that ideal temperature and moisture are available to the seed, optimum planting depth is critical. In regions with dry soils and higher temperatures, deep seed placement ensures optimum temperature and moisture (Mahdi et al., 1998). Deep sowing of seeds also minimizes winter injury and prevents seed damage caused by animals (Brown et al., 2003), however, it delays emergence.

The coleoptile is a sheath that facilitates the emergence of the shoot through the soil crust in monocots. The length of the coleoptile dictates the maximum depth at which seed can be sown. Thus, genotypes with longer coleoptile can be sown deeper to circumvent dry and high-temperature conditions. Whereas genotypes having shorter coleoptiles may fail to emerge if sown too deep and thus result in a poor stand and eventually leading to production losses (Mahdi et al., 1998; Rebetzke et al., 2005; Rebetzke et al., 2007b). Further, an increase in temperature affects coleoptile length negatively. Thus, such genotype*environmental interactions can be devastating on crop yield (Jame and Cutforth, 2004; Rebetzke et al., 2016). Extremely dry situations during the fall season (Budak et al., 1995; Schillinger et al., 1998) and dry spring in the northern Great Plains lead to a poor establishment of hard winter and hard spring wheat, respectively. Extreme fluctuations in weather with changing climate necessitate an adjustment in the breeding programs towards developing crop varieties having longer coleoptiles to ensure better plant stands and establishment.

Present-day wheat varieties' genetic potential for coleoptile length cannot adequately meet the requirements of deep-sowing farming practices and of changing climate. Two reasons responsible for the poor genetic makeup for coleoptile length are; (1) no dedicated breeding effort has been made for improving coleoptile length of wheat varieties; (2) development of semi-dwarf wheat varieties using dwarfing genes *Rht-B1b* and *Rht-D1b* which suppresses or have association with a locus which suppresses coleoptile length (Allan et al., 1962; Allan, 1980; Yu and Bai, 2010; Li et al., 2011; Rebetzke et al., 2016).

Molecular markers linked to genes or quantitative trait loci (QTLs) can facilitate simultaneous marker-assisted breeding and pyramiding for several traits, avoiding laborious and time-consuming phenotyping. Recently, a few QTL mapping studies in spring wheat have mapped several QTLs that control coleoptile length on chromosomes 1A, 1B, 1D, 2B, 2D, 3A, 3B, 3D, 4A, 4BS (*Rht-B1b*), 4DS (*Rht-D1b*), 5A, 5B, 5D, 6A, 6B, and 7B (Rebetzke et al., 2007a; Spielmeyer et al., 2007; Yu and Bai, 2010; Rebetzke et al., 2014; Singh et al., 2015; Li et al., 2017) However, linkage mapping studies have lower power in identifying QTLs with smaller effect and typically demarcate the QTLs to large genomic regions of 15-20 cM (Tuberosa et al., 2002; Korte and Farlow, 2013).

Nearly all previous studies (Spielmeyer et al., 2007; Yu and Bai, 2010; Rebetzke et al., 2014; Singh et al., 2015) consistently mapped QTLs close to *Rht-B1b* and *Rht-D1b*, however, the diverse populations used in those studies led to the identification of distinct novel loci; on chromosomes 1B, 3D, 4DL, and 5AS using a Chinese wheat variety (Yu and Bai, 2010); on chromosomes 1D, 3A, 6A, and 7B using a population derived from Australian cultivars (Spielmeyer et al., 2007; Rebetzke et al., 2014); on chromosomes 3BS and 3BL using Indian cultivars (Singh et al., 2015); and on chromosomes 1BS, 2DS, 4BS, and 5BL using diverse 893 accessions collected from around the world (Li et al., 2017). This suggests that there are a number of QTLs for coleoptile length and therefore, the potential of utilizing these distinct loci in the development of varieties suitable to specific regions.

Genome-wide association (GWAS) is a powerful tool for dissecting genetic architecture of complex traits with the availability of high-density SNP arrays (Wang et al., 2014) and next-generation sequencing technologies (Poland et al., 2012; Ayana et al., 2018; Ramakrishnan et al., 2019; Sidhu et al., 2019). Further, GWAS can effectively identify many natural allelic variations in a large set of unrelated individuals as compared to the traditional QTL mapping (Huang and Han, 2014). Li et al. (2017) conducted GWAS using a global wheat collection of 893 accessions and identified two major QTLs for coleoptile length. These two QTLs are present on chromosome 4B and 4D, independent of *Rht-B1b* and *Rht-D1b* respectively, but their physical locations are unknown. Though a number of QTLs have been mapped in spring wheat and a few in winter wheat, they may not cover the entire variation for coleoptile length. Further, most of the QTLs cover a large genomic region and information on functional characterization of these QTLs is lacking. The functions of candidate genes have only been reported in one study (Singh et al., 2015) where cell wall expansion genes were found in two QTL regions. The functional characterization of genes is necessary to use them efficiently at the molecular and genetic level. Furthermore, understanding the function of genes will also help in navigating the complexity that arises due to breeding for longer coleoptiles, but shorter shoots simultaneously.

Allan et al. (1962) reported the correlation between coleoptile length and final stand establishment in fall sown winter wheat varieties. However, no study has been done to explore the genetic regions controlling coleoptile length in winter wheat varieties of the USA, even though regions of low-precipitation in the Great Plains and Pacific Northwest necessitates deep sowing to ensure moisture for germination (Budak et al., 1995; Schillinger et al., 1998) and better winter survival. Identification and characterization of QTLs by exclusively using winter wheat varieties will shed light on the underlying diversity for coleoptile length, and provide linked markers to facilitate marker-assisted selection. Further, annotation of genes associated with coleoptile length in the candidate regions will help understand the molecular mechanism of coleoptile length in wheat and other monocots.

The objectives of this study were; (i) mapping QTLs that control the length of coleoptile by conducting genome-wide association analysis in a hard winter wheat panel of 298 winter wheat accessions; (ii) identifying SNP markers linked to QTLs for marker-assisted selection; (iii) identifying candidate genes located in the QTL regions.

## Materials and Methods

### Plant Materials

In the present study, we used a hard winter wheat association mapping panel (HWWAMP) of 298 winter wheat accessions developed under the USDA TCAP project (Guttieri et al., 2015). The total collection of 298 accessions consists of released varieties since the 1940s and breeding lines from the US hard winter wheat growing region including Colorado, Kansas, Michigan, Montana, Nebraska, North Dakota, Oklahoma, South Dakota, and Texas. Additional physiological and agronomic data about the HWWAMP accessions is available in the T3/Wheat database (https://triticeaetoolbox.org/wheat/pedigree/pedigree_info.php).

### Experimental Setup

Seed for all 298 HWW accessions were harvested from the field and dried to 11–13% moisture content. The seeds of each line were then carefully cleaned with a Carter Day dockage tester, and clean uniform seeds from the #2 middle sieve were collected for this experiment. Coleoptile lengths of 298 accessions were evaluated in three independent experiments with two replications in each experiment. In each experiment, 10 healthy-looking seeds of each genotype were placed and germinated on a wet paper towel measuring 15 cm x 10 cm (SGB1924B, Anchor Paper Co., USA). Seeds were placed about 1 cm apart with germ end downwards on wet germination paper leaving a 1 cm margin at the bottom. Another wet germination towel of the same size was placed on top. These two germination papers enclosing the seeds were carefully placed in a plastic bag and kept at 4°C for 48 h to break the seed dormancy. Later the plastic bags were hanged vertically in a growth chamber for 14 days at 18°C. After 14 days, coleoptile lengths were measured using a ruler. Distance between the tip of coleoptile and scutellum was considered as the length of coleoptile.

### Data Analysis

The phenotypic data was analyzed using the linear mixed model (LMM) approach, considering all factors as random. The analysis was conducted in R environment (R Core Team, 2016) using R package ‘minque' (Wu, 2014) based on the model:

(1) $$Y_{ijk}=\mu +G_{i}+E_{j}+{GE}_{ij}+R_{i(j)}+e_{ijk}$$

where “*µ*” stands for population mean, “*G*” stands for genotypes, “*E*” for experiments, “*R*” for replications nested under experiments, and “*e*” for the random error. Broad-sense heritability (H^2^) was calculated using equation 2:

(2) $$H^{2}=\frac{\sigma_{G}^{2}}{\sigma_{G}^{2}+\sigma_{E}^{2}/n+\sigma_{G*E}^{2}/nr}$$

Where, σ^2^_*G*_ = *genotype*, σ^2^_*E*_ = *experiment*, σ^2^_G*E_ = genotype * experiment, *r* = *number of replications*, and *n* = *number of experiments*.

### Genotyping

The HWWAMP was genotyped using the wheat Infinium 90K iSelect array (Illumina Inc. San Diego, CA) under the USDA-TCAP (Cavanagh et al., 2013) and the genotypic data (21,555 SNPs) was obtained from the T3 Toolbox (https://triticeaetoolbox.org/wheat/genotyping/display_genotype.php?trial_code=TCAP90K_HWWAMP). To avoid any spurious marker-trait associations, the SNP markers with a minimum allele frequency (MAF) < 0.05 and more than 10% missing SNP data were excluded from further analyses, leaving 15,590 SNP markers. The genetic positions of the wheat Infinium 90K iSelect SNP markers used in the study were obtained from the consensus genetic map of 46,977 SNPs (Wang et al., 2014). The SNP flanking sequences were mapped to wheat Chinese Spring RefSeq v1.1 assembly (IWGSC et al., 2018) using BLASTN to identify the physical location of the mapped SNPs.

### Population Structure And Linkage Disequilibrium

Population structure among the 298 winter wheat accessions was studied to determine any relationship between breeding programs and coleoptile length. We used a set of 15,590 SNP markers with MAF > 0.05 and less than 10% missing genotypic data to estimate the population structure using a model-based Bayesian cluster analysis program, STRUCTURE v2.3.4 (Pritchard et al., 2000). The admixture model was used with 10 independent replicates for each value of genetic groups (*K = 1-10*) followed by 10,000 iterations of burn-in and 10,000 Markov Chain Monte Carlo (MCMC) iterations. Structure Harvester (Earl and vonHoldt, 2012) was used to extract the output of the structure analysis. The optimum number of clusters was inferred using statistic ΔK (delta K) (Evanno et al., 2005), which is based on the rate of change in the log probability of given data, between successive K values. Furthermore, we conducted principal component analysis (PCA) in TASSEL 5.0 (Bradbury et al., 2007) using the same set of markers and used the PCA covariates for GWAS analysis. Linkage disequilibrium (LD) decay distances for the HWWAMP were calculated using TASSEL v5.0 (Bradbury et al., 2007) with only 1,842 markers taking out non-informative markers in our previous study (Ayana et al., 2018). The estimated r^2^ values were plotted against the genetic distance (cM) to elucidate the LD decay for all as well as individual genomes. The LD (r^2^ > 0.1) decay distance of about 4.5 cM was estimated for the whole genome (Ayana et al., 2018).

### Marker Trait Associations

Genome-wide association mapping was conducted using 15,590 SNPs and coleoptile data from 298 HWWAMP accessions using the mixed linear model (MLM) (Yu et al., 2006) implemented in TASSEL (Trait Analysis by association, Evolution, and Linkage) v 5.0 software (Bradbury et al., 2007). MLM is mathematically represented as:

(3) y=Xβ+Zu+e

where *y* represents the vector of the phenotypic values, *β* represents fixed effects due to the marker and population structure, *u* represents the vector of the random effects, *e* represents the vector of residuals, and *X* and *Z* are the incidence matrices for *β* and *u*, respectively.

MLM was used as it incorporates kinship and population structure as covariates to minimize the confounding effects, reducing the probability of type-I error when compared to the general linear model (GLM). Kinship (K) was estimated using the Centered IBS (identity by state) method in TASSEL v 5.0 (Endelman and Jannink, 2012). By default, TASSEL v5.0 uses PCA as covariates to adjust for the population stratification. We incorporated the first four PCAs as covariates in the MLM model to reduce the confounding effects. As the false discovery rate (FDR) correction for multiple testing was too stringent, markers with a −log10(p-value) > 3 were considered as significant associations. Furthermore, MLM results from TASSEL v5.0 were confirmed using MLM and SUPER in the genome association and prediction integrated tool (GAPIT) (Lipka et al., 2012) implemented in the R environment (R Core Team, 2016). Further, the identified QTLs were also subjected to five-fold validation (Ramakrishnan et al., 2019). Briefly, the population was randomly divided into five subsets of equal size and process was repeated five times. Out of each of the five subsets, four (240 lines) were used for marker-trait association analysis and the last set (60 lines) was used to cross-validate the significant markers using t-test among different alleles of each significant SNP marker.

### Identification and Annotation of the Candidate Genes in the QTL Regions

We used the flanking sequence of significant SNPs to physically map them on Chinese Spring Refseqv1.1 (IWGSC, 2018) using BLASTN search with an E-value cut off 1e^-50^. To demarcate the candidate QTL regions, the SNP markers with *P < 0.005,* both up- and downstream of the most significant marker, were identified. The coding sequences (CDS) of high confidence genes (https://urgi.versailles.inra.fr/jbrowseiwgsc) from each of these QTL regions were extracted in the FASTA format and Blast2Go software (https://www.blast2go.com) was used for functional gene annotation. Consequently, we identified the candidate genes that may be associated with coleoptile length based on the LD Decay in the region (Ayana et al., 2018) and their putative functions after a thorough review of the literature.

## Results

### Phenotypic Variance

**Figure 1:**
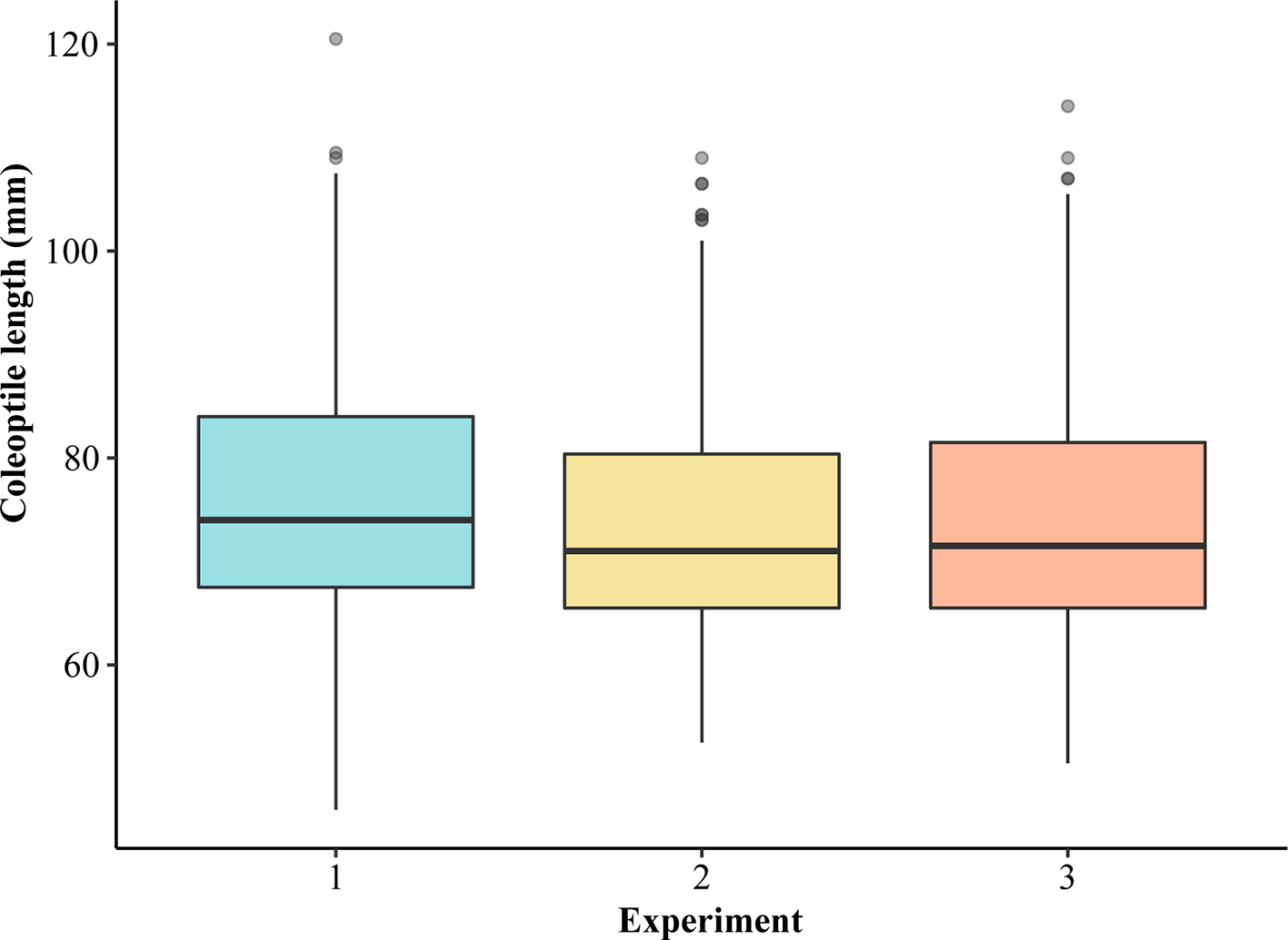
Boxplots showing the distribution of average coleoptile length of 298 genotypes of hard winter wheat association mapping panel (HWWAMP) in three experiments.

Coleoptile length within 298 winter wheat accessions varied from 49.40 to 111.00 mm with an overall mean of 74.65 mm (**Supplementary Table S1**). LMM analyses revealed that the three experiments were consistent (**Figure 1**, **Supplementary Table S2**). Average coleoptile length for the three independent experiments (further referred to as Exp1, Exp2, and Exp3) was 76.10, 73.50, and 74.00 mm, respectively (**Figure 1**). Overall, only 1.24% of the variation was contributed by experiments and replications together. The estimated broad-sense heritability for coleoptile length was 73.4%. The median coleoptile length was 71.75 mm. About 25% of the genotypes were less than 66.33 mm and 25% were above 81.17 mm. The majority of the genotypes in all the experiments reached a coleoptile length of ≥ 65 and ≤ 70 mm (**Figure 1**). An accession from Oklahoma ‘OK05723W’ had the shortest coleoptile (49.40 mm) while the cultivar ‘AGATE’ had the longest coleoptile (111.00 mm). We also evaluated if the seed source (location) may have an impact on the coleoptile length by comparing the coleoptile length of two varieties from four different locations. The genotype and location effects were found to be significant for two genotypes. However, genotype*location interaction was non-significant, with the ranking of two varieties being the same across four locations. Thus, the growing environment did not significantly impact the ranking of the genotypes for coleoptile length.

### LD Analysis and Population Structure

The hard winter wheat association-mapping panel was characterized for LD in our previous study (Ayana et al., 2018). LD decay was calculated based on the r^2^ values for the whole genome and within each genome of the association panel. The distance where LD value (r^2^) decreases below 0.1 or half strength of D' (D' = 0.5) was estimated based on the curve of the nonlinear logarithmic trend line. LD dropped to 0.5 at about 4.5 cM for whole-genome; whereas, LD extent in A and B and D genomes was around 3.4 and 3.6 cM, but much larger in D genome (14.2 cM) owing to fewer markers.

The association-mapping panel used in this study is comprised of 298 winter wheat cultivars/breeding lines from different regions of the USA. We investigated the population structure to reveal if the association-mapping panel is structured, based on the breeding programs/origin; and figure out any relationship of structure with the coleoptile length. We identified four sub-populations in the HWWAMP, namely: P1, P2, P3, and P4 (**Supplementary Figure S1**). Populations P1, P2, P3, and P4 consist of 120, 34, 33, and 111 genotypes, respectively with a corresponding average coleoptile length of 79.13, 75.18, 69.91, and 72.20 mm. The average coleoptile length of population P1 was higher than the populations P2, P3, and P4; however, it was statistically different only from P3 and P4 (**Supplementary Table S3**).

### Marker Trait Associations (MTAs)

**Figure 2:**
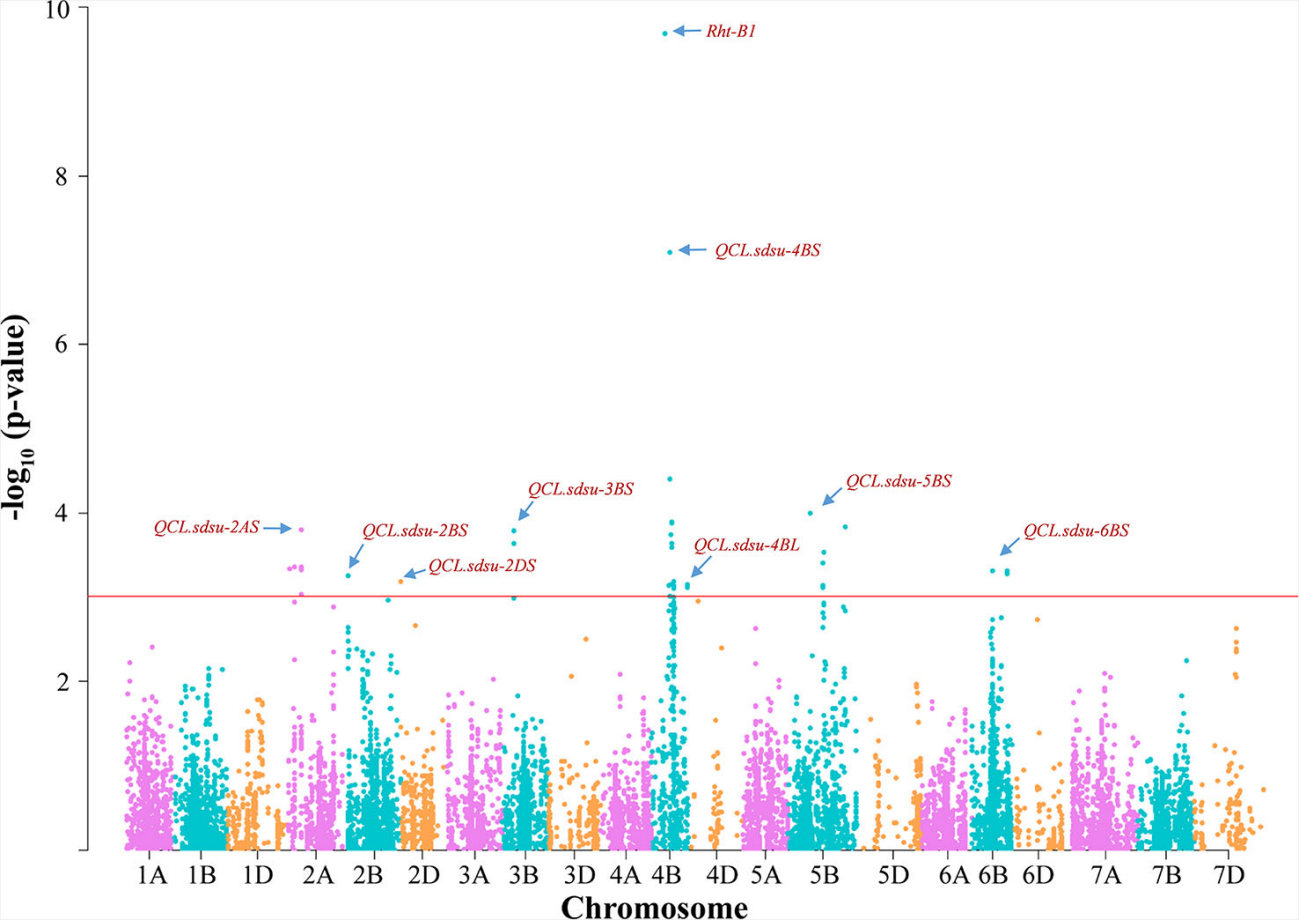
Distribution of marker-trait associations for coleoptile length in hard winter wheat association mapping panel (HWWAMP) based on their –log(10) p-values. Manhattan plot was developed using a mixed linear model (MLM) in TASSEL v.5. The -log10 (p-values) from a genome-wide scan are plotted against particular position on each of the 21 wheat chromosomes. Horizontal line indicate genome-wide significance thresholds.

In total, GWAS analysis using MLM in TASSEL v5.0 identified 46 significant SNPs (*P < 0.001*) in nine genomic regions present on seven different chromosomes (**Supplementary Table S4**). Based on the threshold value of –log10 (p-value) > 3, we identified 14, 1, 1, 2, 18, 6, and 4 significant SNPs on chromosomes 2A (*QCL.sdsu-2AS*), 2B (*QCL.sdsu-2BS*), 2D (*QCL.sdsu-2DS*), 3B (*QCL.sdsu-3BS*), 4B (*QCL.sdsu-4BS* and *QCL.sdsu-4BL*), 5B (*QCL.sdsu-5BL*), and 6B (*QCL.sdsu-6BL*), respectively (**Figure 2**). Like previous studies (Rebetzke et al., 2007a; Rebetzke et al., 2014; Li et al., 2017), we also found *Rht-B1*, a Gibberelin (GA) insensitive dwarf allele to be associated with coleoptile length. Out of 298 genotypes, 201 (67.4%) carried the dwarf allele (allele 2) and 84 (28.2%) carried the tall allele (allele 1) of *Rht-B1*. In the current study, *Rht-B1* linked SNP was highly significant with a -log_10_ (p-value) of 9.69 and explained 16.7% of the variation. The average coleoptile length of genotypes carrying allele 1 of *Rht-B1* was 13.50 mm longer than genotypes carrying allele 2. Another dwarfing gene, *Rht-D1*, was not found to be associated with coleoptile length in the current study as only 14 (4.7%) of 298 individuals carried the dwarf allele for this gene.

In total, the eight QTLs, in addition to *Rht-B1* explained 42.2% of variation in coleoptile length (**Table 1**). After *Rht-B1, QCL.sdsu-4BS* explained the highest variation (10.6%), followed by *QCL.sdsu-5BL and QCL.sdsu-2AS*, explaining 5.26% and 5.00% variation, respectively. The most significant SNPs linked to QTLs, *QCL.sdsu-2AS*, *QCL.sdsu-2BS*, *QCL.sdsu-2DS*, *QCL.sdsu-3BS*, *QCL.sdsu-4BS*, *QCL.sdsu-4BL*, *QCL.sdsu-5BL,* and *QCL.sdsu-6BL,* were *D_F1BEJMU02JILPD_53, BS00067280_51, D_contig17313_245, Tdurum_contig43252_1407, IAAV971, RAC875_rep_c82932_407, Tdurum_contig67535_391,* and *BS00065357_51*, respectively (**Table 1**). All eight QTLs identified using TASSEL v5.0 were validated using MLM (P+K model) and SUPER algorithms implemented in GAPIT to further ascertain the significance. However, the QQ plots from different algorithms revealed that MLM model has the better fit than SUPER (results not shown).

**Table 1:** Most significant SNP markers linked to the eight QTLs for coleoptile length detected from genome-wide association analysis of 298 winter wheat genotypes.

QTL	Marker	Chromosome	Mb^a^	-log_10_(p-value)	R^2^ (%)	T-test^b^
*QCL.sdsu-2AS*	D_F1BEJMU02JILPD_53	2A	15.61	3.80	5.00	6.64E-03
*QCL.sdsu-2BS*	BS00067280_51	2B	6.10	3.25	4.10	6.76E-02
*QCL.sdsu-2DS*	D_contig17313_245	2D	93.44	3.18	4.15	1.78E-05
*QCL.sdsu-3BS*	Tdurum_contig43252_1407	3B	23.78	3.79	5.03	2.74E-04
*QCL.sdsu-4BS*	IAAV971	4B	40.75	7.10	10.56	1.15E-06
*QCL.sdsu-4BL*	RAC875_rep_c82932_407	4B	666.04	3.14	3.93	1.45E-03
*QCL.sdsu-5BL*	Tdurum_contig67535_391	5B	536.63	4.00	5.26	5.18E-02
*QCL.sdsu-6BS*	BS00065357_51	6B	705.75	3.31	4.19	1.25E-01
*Rht-B1*	*Rht-B1*	4B	30.86	9.69	16.69	–

a The SNP position (Mb) is based on the CS RefSeq v1.1 (IWGSC, 2018).

b *P-value* obtained from the 5-fold cross validation.

In addition, five-fold cross-validation was used to ascertain the significance of the identified SNP markers in each genomic region. After dividing the HWWAMP into five subsets, we used four sets for the marker-trait association and the remaining set of 60 accessions were used for cross-validation of significant markers. The cross-validation confirmed that six SNPs linked to QTLs, *QCL.sdsu-2AS, QCL.sdsu-2DS, QCL.sdsu-3BS, QCL.sdsu-4BS, QCL.sdsu-4BL, and QCL.sdsu-5BL,* were significantly associated with coleoptile length (Based on p-value for T-test, **Table 1**). Another QTL, *QCL.sdsu-2BS* had p-value of 0.06 from the respective t-test; thus, marginally out at 5% level of significance.

**Figure 3:**
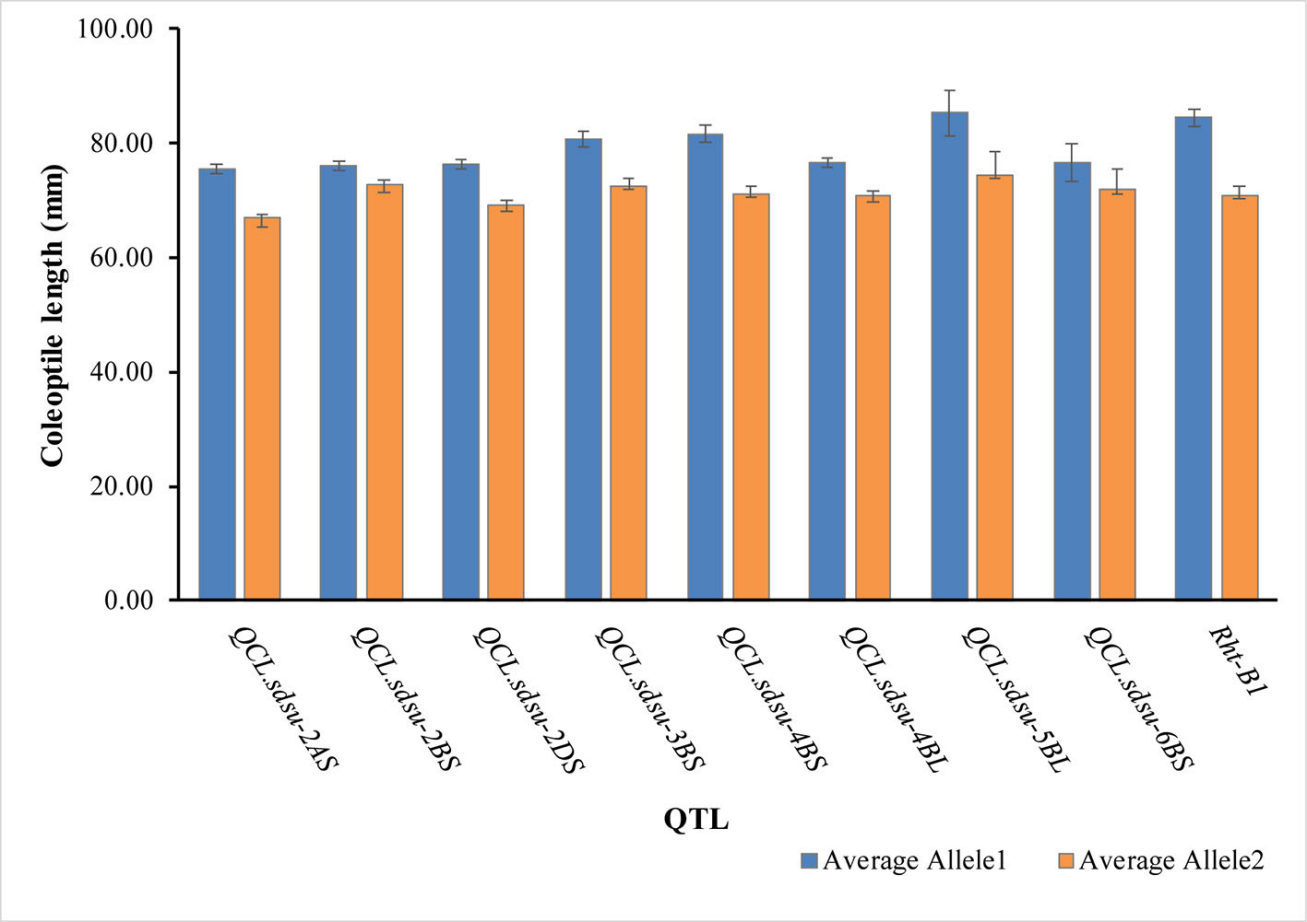
Average coleoptile length of hard winter wheat association mapping panel genotypes corresponding to each allele of the most significant marker on the respective chromosome. Error bars are also shown at top of the bars.

Pairwise comparison among the alleles of the significant SNPs also verified their association with coleoptile length (**Figure 3**, **Supplementary Table S5**). Positive allele (allele 1) increases the coleoptile length and its counterpart, negative allele (allele 2) decreases the coleoptile length. Allele 1 and allele 2 for each of the most significant SNP on each chromosome is given in **Supplementary Table S5**. Individually, coleoptile length difference between the allele 1 and allele 2 of the SNP on chromosomes 2A, 2B, 2D, 3B, 4BS, 4BL, 5B, and 6B was 8.62, 3.51, 7.13, 8.25, 10.70, 5.76, 10.94, and 4.56 mm, respectively. All the differences were significant at a p-value < 0.05. Overall, *QCL.sdsu-5BL* has the largest substitution effect (10.94 mm) for coleoptile length following *Rht-B1*.

### Genotypes With Longer Coleoptiles

We found eight genotypes with coleoptile length longer than 100 mm, namely: ‘CRIMSON’, ‘SCOUT66’, ‘GENOU’, ‘KIRWIN’, ‘KAW61’, ‘LONGHORN’, ‘MT06013’, and ‘AGATE’ (**Table 2**, **Supplementary Table S6**). ‘AGATE’ had the longest coleoptile length (average 111 mm) followed by ‘MT06103’ (average 110.6 mm). Interestingly, ‘MT06103’ carried positive alleles (allele 1) for all the SNPs except *Rht-B1*. ‘AGATE’ was positive for all the SNPs. Significant SNP data for the other six genotypes are given in **Table 2**. From the perspective of most significant SNPs, all of the eight genotypes with the longest coleoptiles carried positive alleles for SNPs on chromosomes 2A, 2B, 4B, and 6B. On the contrary, SNP “Tdurum_cotig67535_391” on chromosome 5B was only positive in ‘GENOU’, ‘AGATE’, and ‘MT06103’.

**Table 2:** Hard winter wheat association mapping panel (HWWAMP) genotypes with coleoptile length longer than 100 mm, along with their genotype for the most significant markers related to coleoptile length.

SNP on	2A	2B	2D	3B	4BS	4BL	5B	6B		*Rht-B1*	CL*	CSE‡
Substitution effect	8.6	3.5	7.1	8.2	10.7	5.8	10.9	4.6		13.5		
Allele 1/Allele 2	C/T	T/C	C/A	T/C	C/T	A/G	C/A	C/T		a/b		
CRIMSON	1	1	1	1	1	2	2	1		1	101.00	56.27
SCOUT66	1	1	1	1	1	1	2	1		1	101.50	62.04
GENOU	1	1	N	2	1	1	1	1		1	101.80	57.59
KIRWIN	1	1	1	1	1	1	2	1		1	103.66	62.06
KAW61	1	1	1	2	1	1	2	1		1	105.50	53.78
LONGHORN	1	1	1	1	1	1	2	1		1	106.83	62.04
MT06103	1	1	1	1	1	1	1	1		2	110.66	59.48
AGATE	1	1	1	1	1	1	1	1		1	111.00	72.98

*Coleoptile length (mm), ‡Cumulative substitution effect. ‘1' represents positive allele and ‘2' represents the negative allele.

### Identification of Candidate Genes and Putative Functions

To facilitate the identification of candidate genes governing coleoptile length, the chromosome regions were first delimited based on the consensus genetic map (Wang et al., 2014) and LD decay distance from our previous study (Ayana et al., 2018). Subsequently, these demarcated regions were identified by BLASTN, searching the flanking sequence of significant SNPs against CS RefSeqv1.1 (IWGSC, 2018). We then delimited the QTLs region to a 5.3, 5.9, 7, 2, 5.5, and 1.6 Mb region on chromosomes 2AS, 3BS, 4BS, 4BL, 5BL, and 6BL, respectively. Contrarily, the significant markers on chromosomes 2BS and 2DS were localized on the terminal regions of respective chromosomes, with no flanking marker available on the terminal end in the consensus genetic map (Wang et al., 2014). Therefore, the terminal regions, 6.9 and 10.3 Mb from 1bp extending up to the flanking marker on the distal end were identified as a candidate region on chromosome 2BS and 2DS, respectively. The putative genes from these regions were further narrowed down based on the LD decay distance and proximity to the most significant SNP. Finally, we annotated the coding sequences of high confidence (HC) genes in these candidate regions using the Blast2Go (Conesa et al., 2005).

Overall, 825 high confidence genes from the eight candidate regions were annotated. Among these genes, we identified candidate genes with possible involvement in coleoptile length based on proximity to the most significant SNP and a thorough review of the literature. Accordingly, we found 28 genes predicted to encode 10 different putative proteins that can play a role in governing the coleoptile length (**Table 3**). In the 5.3 Mb region spanning *QCL.sdsu-2AS*, we found five genes that encode 1-aminocyclopropane-1-carboxylate oxidase homolog 1-like protein, which have possible involvement in coleoptile length. Another gene, *TraesCS2A02G033900*, is predicted to have a jacalin-like lectin domain, found to be a coleoptile specific lectin in barley (Grunwald et al., 2007). For QTL *QCL.sdsu-2BS*, we identified two genes encoding a cytochrome P450 87A3-like, and a probable indole-3-pyruvate monooxygenase *YUCCA5-* like proteins. Similarly, two different genes were identified in the region harboring *QCL.sdsu-2DS* encoding for the same two protein. The 2DS region also harbors four other genes predicted to encode cytochrome P450 85A1-like proteins. In these two regions (2BS and 2DS), genes encoding cytochrome P450 87A3-like and cytochrome P450 85A1-like proteins are of specific interest-based on their established role in other species. Another QTL, *QCL.sdsu-3BS* in the 5.9 Mb region of chromosome 3BS harbored 10 genes of specific interest, all predicted to encode an expansin-like protein. The fifth QTL, *QCL.sdsu-4BS* was delimited to a 7 Mb region with 65 annotated genes including two genes of interest viz. *TraesCS4B02G052000* and *TraesCS4B02G049800* putatively encoding phytochrome A-like and receptor protein kinase *TMK1*-like proteins, respectively. In the region harboring *QCL.sdsu-4BL*, a gene annotated as putative 2-oxoglutarate-dependent dioxygenase seems a likely candidate as it catalyzes several metabolic pathways in plants such as a gibberellins pathway. Most of the identified genes from the *QCL.sdsu-5BL* region were annotated as “predicted proteins”, with no clear differentiation into protein families. Thus, only one gene with a likely role in coleoptile length was discovered in a 5.5 Mb region harboring this novel QTL (**Table 3**). Further, we were unable to select any candidate genes in the region harboring QTL *QCL.sdsu-6BS* based on the available literature.

**Table 3:** Annotation of candidate genes in the demarcated QTL regions identified through GWAS in hard winter wheat association mapping panel (HWWAMP).

Chr	QTL	Gene ID^a^	Start position of the gene (bp)^a^	Gene Annotation
2AS	*QCL.sdsu-2AS*	*TraesCS2A02G025800*	12,129,444	1-aminocyclopropane-1-carboxylate oxidase homolog 1-like
		*TraesCS2A02G025900*	12,139,588	1-aminocyclopropane-1-carboxylate oxidase homolog 1-like
		*TraesCS2A02G026500*	12,247,082	1-aminocyclopropane-1-carboxylate oxidase homolog 1-like
		*TraesCS2A02G036900*	15,756,318	1-aminocyclopropane-1-carboxylate oxidase homolog 1-like
		*TraesCS2A02G037900*	15,959,789	1-aminocyclopropane-1-carboxylate oxidase homolog 1-like
		*TraesCS2A02G033900*	15,011,079	mannose/glucose-specific jacalin-like lectin
2BS	*QCL.sdsu-2BS*	*TraesCS2B02G009100*	5,041,094	cytochrome P450 87A3-like
		*TraesCS2B02G010100*	5,628,213	probable indole-3-pyruvate monooxygenase YUCCA5
2DS	*QCL.sdsu-2DS*	*TraesCS2D02G012100*	5,747,458	probable indole-3-pyruvate monooxygenase YUCCA5
		*TraesCS2D02G012800*	6,204,775	cytochrome P450 87A3
		*TraesCS2D02G014400*	7,062,903	cytochrome P450 85A1
		*TraesCS2D02G014500*	7,072,238	cytochrome P450 85A1
		*TraesCS2D02G014600*	7,085,341	cytochrome P450 85A1
		*TraesCS2D02G014700*	7,089,687	cytochrome P450 85A1
3BS	*QCL.sdsu-3BS*	*TraesCS3B01G051000*	25,906,973	expansin
		*TraesCS3B01G051100*	25,921,029	expansin
		*TraesCS3B01G051200*	26,043,431	expansin
		*TraesCS3B01G051300*	26,057,175	expansin
		*TraesCS3B01G051400*	26,191,126	expansin
		*TraesCS3B01G051500*	26,246,150	expansin
		*TraesCS3B01G051600*	26,301,286	expansin
		*TraesCS3B01G051800*	26,385,625	expansin
		*TraesCS3B01G051900*	26,399,446	expansin
		*TraesCS3B01G052000*	26,430,002	expansin
4BS	*QCL.sdsu-4BS*	*TraesCS4B02G052000*	40,780,124	phytochrome A
		*TraesCS4B02G049800*	38,280,457	receptor protein kinase TMK1-like
4BL	*QCL.sdsu-4BL*	*TraesCS4B02G389500*	665,956,360	putative 2-oxoglutarate-dependent dioxygenase
5BL	*QCL.sdsu-5BL*	*TraesCS5B02G356700*	536,321,998	auxin Efflux Carrier family protein isoform X1

a Gene ID and physical positions are based on CS RefSeq v1.1 (IWGSC, 2018).

## Discussion

### Breeding Wheat for Longer Coleoptiles

Winter wheat is grown in a range of harsh environments around the globe, (Stockton et al., 1996; Bai et al., 2004) and challenges are further elevated by rising temperatures and unpredictable droughts. In conditions like hard and dry grounds (drought), and unpredicted freezing and thawing, early wheat establishment is challenged, potentially leading to lower yields (Stockton et al., 1996; Bai et al., 2004). One of the solutions to increase seedling establishment is deep sowing in order to exploit the leaching moisture regime. Coleoptile length is the limiting factor for deep planting since it affects the emergence capacity of seedlings planted deep, especially in fields with thicker stubble (No-till) and/or crusted soil surfaces (Rebetzke et al., 2014). Furthermore, around 90% of the modern semi-dwarf wheat varieties have GA-insensitive dwarfing genes, which are strongly associated with shorter coleoptiles (Rebetzke et al., 1999; Li et al., 2017; Grover et al., 2018). One of the easier ways to increase coleoptile length is pyramiding of larger effect QTLs in modern-day wheat cultivars. A number of studies have shown that coleoptile length is under strong additive gene control (Rebetzke et al., 2007a; Spielmeyer et al., 2007; Yu and Bai, 2010; Li et al., 2011; Rebetzke et al., 2014; Singh et al., 2015; Li et al., 2017), thus identification of novel QTLs for increased coleoptile length would be desirable. Moreover, limited information is available in winter wheat, compelling winter wheat breeders to rely on spring wheat resources. Accordingly, we employed GWAS using 298 hard winter wheat lines in this study to develop resources for longer coleoptile length in winter wheat.

### Phenotypic Evaluation for Coleoptile Length

Our results for phenotypic evaluation show that sufficient variation for coleoptile length exists in the hard winter wheat association panel, with coleoptile length ranging from 49.4 to 111 mm which overlaps with previous studies; 25 to 170 mm (Rebetzke et al., 2014) and 57 to 202 mm (Li et al., 2017). Variations among the ranges in different studies can be attributed to the diversity among the lines used and the temperature at which seedlings were grown. HWWAMP constitutes of released winter wheat cultivars and breeding lines from US winter wheat breeding programs; however, more diverse germplasm was evaluated in other studies (Rebetzke et al., 2014; Li et al., 2017). The average coleoptile length of lines from the South Dakota breeding program was highest, whereas, lines from the Michigan breeding program had the shortest coleoptile, but we did not see any significant differences among any of the breeding programs. This suggests that there is no specific focus or indirect selection for coleoptile length in any of the hard winter wheat breeding programs in the US.

Plant height has been known to be correlated with be the coleoptile length (Allan et al., 1962; Allan, 1980; Yu and Bai, 2010; Li et al., 2011; Rebetzke et al., 2016). Although we did not collect the plant height data on 298 accessions for this experiment, the HWWAMP has been evaluated for agronomic traits including plant height under the USDA-NIFA TCAP grant at several locations and the data is available in the wheat T3 database. We compared plant height at four locations to the coleoptile length of 298 accessions in this study. As expected, plant height and coleoptile length showed correlation (0.28, 0.30, 0.26, and 0.37 for four locations, respectively), but these correlations were not very high. This suggests that other factors (genomic regions) in addition to plant height QTLs identified in this study affect the coleoptile length.

### QTLs for Coleoptile Length

In the present study, MLM based genome wide associations identified eight QTLs associated with coleoptile length on seven different chromosomes. The identified QTLs were validated using five-fold cross-validation (Ramakrishnan et al., 2019). This approach validated six of the eight identified QTLs, namely *QCL.sdsu-2AS, QCL.sdsu-2DS, QCL.sdsu-3BS, QCL.sdsu-4BS, QCL.sdsu-4BL, and QCL.sdsu-5BL* (**Table 1**). Another QTL, *QCL.sdsu-2BS* and *QCL.sdsu-6BL* were not validated using the five-fold approach. These could be potential associations affecting coleoptile length and need further validation.

**Figure 4:**
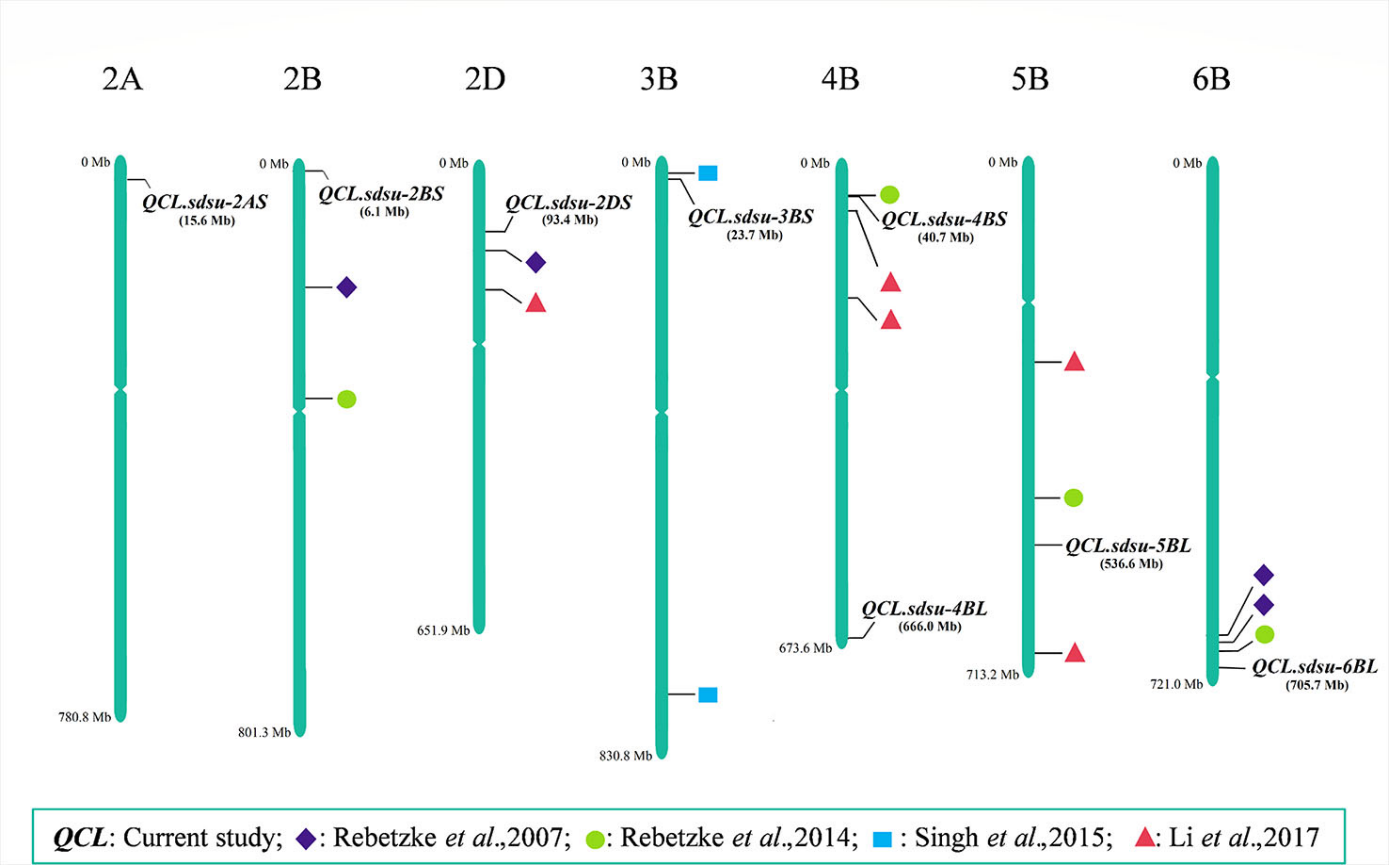
Chromosomal positions of QTLs associated with coleoptile length identified in the current study and their comparative analysis with the previous studies. The scale represents physical distance based on Chinese Spring RefSeq 1.1 (IWGSC, 2018).

We compared the findings of this study by fetching the physical location of previously reported QTLs from several coleoptile length mapping studies (Rebetzke et al., 2007a; Rebetzke et al., 2014; Singh et al., 2015; Li et al., 2017) (**Figure 4**). As a result, we identified three novel QTLs, namely, *QCL.sdsu-2AS*, *QCL.sdsu-4BL*, and *QCL.sdsu-5BL* and four QTLs that are in the proximity to previously mapped QTLs (**Figure 4**). Among the novel QTLs, *QCL.sdsu-5BL* explains largest variation (R^2^ = 5.26%) followed by *QCL.sdsu-2AS* (R^2^ = 5.00%). Furthermore, the pairwise comparison among the alleles of the significant SNPs revealed that *QCL.sdsu-5BL* has the largest substitution effect after *Rht-B1*. Therefore, *QCL.sdsu-5BL* is a valuable novel QTL which could be used to compensate for negative effect of *Rht-B1* locus on coleoptile length.

Two QTLs namely *QCL.sdsu-2DS* and *QCL.sdsu-3BS*, previously mapped using Simple sequence repeats (SSR) markers (Rebetzke et al., 2007b; Singh et al., 2015) were also validated using SNPs in this study. The newer positions of these two QTLs are likely more accurate as highly saturated SNP markers were used in the current study compared to less dense SSR markers used in the previous studies. Different studies (Rebetzke et al., 2014; Li et al., 2017) have reported a QTL for coleoptile length on chromosome 4BS. In this study, we identified a QTL (*QCL.sdsu-4BS*) in the same region, which is around 10 Mb apart from the *Rht-B1* gene (IWGSC, 2018). Based on the estimated LD (r^2^ = 0.54) between the *Rht-B1* and *QCL.sdsu-4BS*, these two could be different regions or *QCL.sdsu-4BS* could likely represent *Rht-B1*. Further investigation is needed to validate the independence of these regions.

Out of the nine significant associations (including *Rht-B1*) found in the current study, seven are mapped to the B genome. Furthermore, among the total unique QTLs mapped for coleoptile length so far (including this study), 57% QTLs are mapped on the B genome, 26% QTLs are mapped on the D genome and 17% QTLs are mapped on the A genome. Thus, it seems that B genome comparatively may have more genes controlling the coleoptile length. It would be interesting to study the variation among the diploid progenitors of wheat for coleoptile length.

**Figure 5:**
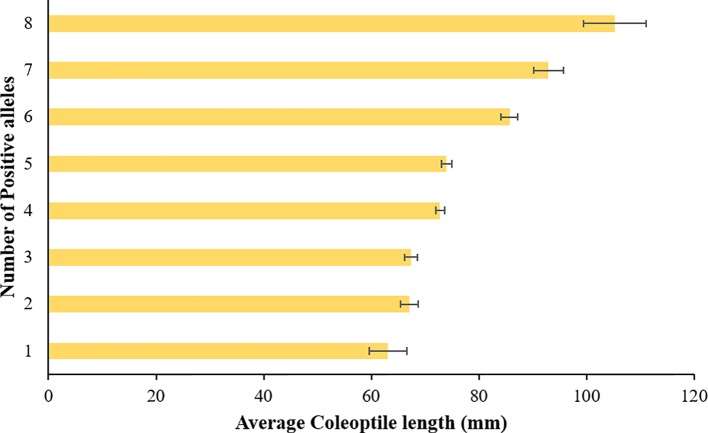
Average coleoptile length corresponding to each stack of positive alleles in hard winter wheat association mapping panel (HWWAMP).

Pyramiding of favorable QTLs can be successfully used for developing varieties with longer coleoptile (Li et al., 2017). In agreement with the previous studies (Rebetzke et al., 2014; Li et al., 2017), we observed an additive effect for coleoptile length among the identified QTLs in the current study. The stacking of positive alleles at different loci increased coleoptile length in additive fashion (**Figure 5**). A cultivar ‘AGATE’ has all the positive alleles for associated SNPs and has the longest coleoptile length. We also compared the allelic composition of three cultivars having shortest coleoptile length. These three cultivars namely ‘GARRISON’, ‘OK5723W’, and ‘OK04505’ have negative alleles (allele 2) at six, five, and four associated SNPs, respectively. In addition, all three cultivars have the dwarfing allele for *Rht-B1*. Though, it will be desirable to keep the negative allele of *Rht-B1* so that the stature/height of cultivars remains semi-dwarf. We identified a breeding line ‘MT06103’ which has the positive alleles at all loci except for the *Rht-B1*. MT06103 has coleoptile length very close to ‘AGATE’ (**Table 2**). While studying the seedling emergence in fall sown wheat, Allan et al., 1962 also found a selection (14 X 50-3 B-4), which was moderately short in plant height but was ranked towards top with respect to coleoptile length. Thus, it is evident that coleoptile length can be improved while maintaining short stature of plant. Thus, such genotypes which already have all the favorable alleles can directly be exploited in winter wheat breeding programs to improve the coleoptile length of the new cultivars.

### *In silico* Gene Annotation of the Candidate Regions

After a thorough examination of the available literature and proximity to the most significant SNPs, we identified 27 genes predicted to have a role that could likely affect coleoptile elongation (**Table 3**). We found genes with diverse functions, including phytohormone biosynthesis-related, cytochrome P450 family genes, expansins, etc. that are probable candidates. Further, it is expected that the genes common to many QTL regions are more likely to play a role in determining the length of coleoptile.

Phytohormones are the signaling molecules, which play a crucial role in the development and physiological processes in plants (Rudnicka et al., 2019). Specifically, auxins are a major group of phytohormones, which affect coleoptile length in grass species by inducing cell elongation either directly (Vanneste and Friml, 2009; Paque and Weijers, 2016), or by interacting with other plant hormones such as ethylene (Woodward and Bartel, 2005). Two genes from different candidate regions on chromosomes 2BS and 2DS were predicted as indole-3-pyruvate monooxygenase *YUCCA5* protein, which catalyzes the biosynthesis of indole-acetic acid (IAA), the most commonly occurring natural auxin, from tryptophan (Won et al., 2011). We also found a *PIN* protein (a component of auxin-efflux carrier family) in the *QCL.sdsu-5BL* region. The *PIN* proteins are known to play role in auxin transport and expressed in several plant tissues, affecting plant growth (Zhou and Luo, 2018). Whereas, another putative *ACO1*-like protein was found in the 2AS candidate region. *ACO1*-like protein is a part of the ethylene biosynthetic pathway and is speculated to affect rice coleoptile elongation in stress conditions (Hsu and Tung, 2017).

Brassinosteroids (BRs) play an important role in cell elongation and proliferation (Nakaya et al., 2002), and thus in determining plant height. A BR-deficient (*brd*) mutant was used to characterize *OsDWARF* gene in rice, an orthologue of the tomato *DWARF* gene and *CYP85A1* or *BR6OX1* in Arabidopsis (Shimada et al., 2001; Shimada et al., 2003) and found to affect polar elongation of stem cells (Hong et al., 2002). Another cytochrome P450 superfamily protein *CYP87A3* has been characterized in rice as an auxin-induced gene specifically expressed in coleoptiles (Chaban et al., 2003). In our study, we found putative cytochrome P450 85A1-like and cytochrome P450 87A3 proteins spanning the QTLs, *QCL.sdsu-2BS*, and *QCL.sdsu-2DS* which may affect coleoptile length in wheat. Additionally we found 10 genes all encoding putative expansin proteins in the genomic region spanning *QCL.sdsu-3BS*. Our finding corroborates with Singh et al. (2015) who also reported the presence of expansin like genes in this region while mapping coleoptile length in a biparental mapping population. Expansins have been reported to affect cell growth and elongation (Marowa et al., 2016); and express in wheat coleoptiles and correlate with the coleoptile growth (Gao et al., 2007; Gao et al., 2008). The cytochrome P450 superfamily genes and expansins are thus strong candidates for coleoptile length and need further investigation in wheat.

Further, phytochrome A (*PHY A*) protein identified in the *QCL.sdsu-4BS* candidate region is of specific importance with respect to coleoptile length. In rice, phytochrome A gene is well known to affect coleoptile elongation, plant height, and internode elongation either directly or by affecting jasmonate signaling genes (Garg et al., 2006; Riemann et al., 2008). Apart from these genes, we also found jacalin-like lectin, related to Horcolin protein specifically expressed in barley coleoptiles (Grunwald et al., 2007) and putative 2-oxoglutarate-dependent dioxygenase (**Table 3**), related to a versatile enzyme family catalyzing biosynthesis and catabolism of auxins and gibberellins (Farrow and Facchini, 2014).

## Conclusion

Coleoptile length is regularly evaluated in advanced breeding lines in several breeding programs. However, due to limited knowledge about the underlying QTLs and linked molecular markers, breeding for coleoptile length becomes challenging. Characterization of eight QTLs associated with coleoptile length in winter wheat and identification of tightly linked SNPs could be a valuable resource for wheat breeders. The critical SNPs identified in our study could be used to develop breeder friendly kompetitive allele-specific PCR (KASP) assays (**Supplementary Table S7**) for marker-assisted selection (Rasheed et al., 2016; Gill et al., 2019). Marker-assisted stacking of these QTLs would result in the development of wheat varieties with longer coleoptile. Also, these QTLs can be effectively combined with previously reported QTLs to breed for desired coleoptile length in wheat. In addition, these markers could be weighted and incorporated into the genomic selection strategy. Further functional genomic studies are crucial to validate the effect of the identified candidate genes on coleoptile length.

## Data Availability Statement

All datasets generated for this study are included in the article/**Supplementary Material**. Additional physiological and agronomic data about the HWWAMP accessions is available in the T3/Wheat database (https://triticeaetoolbox.org/wheat/pedigree/pedigree_info.php).

## Author Contributions

JS and SS conceptualized the experiment and designed the methodology. JS, DS, YQ, JH, RT performed the investigation. JS, DS, HG, and NB performed the data analysis. HG, JS, DS, NB, and SS wrote the original manuscript. JH, YQ, RT, and BT contributed to the interpretation of results and revision of the manuscript. All authors approved the manuscript.

## Funding

This project was collectively funded by the USDA hatch projects SD00H538-15 and SD00H695-20 and the Agriculture and Food Research Initiative Competitive Grants 2011-68002-30029 (Triticeae-CAP), 2017-67007-25939 (Wheat-CAP), and 2019-67013-29015 from the USDA National Institute of Food and Agriculture and South Dakota Wheat Commission grant 3X9267. The funders had no role in the study design, data collection, and analysis, decision to publish, or preparation of the manuscript.

## Conflict of Interest

The authors declare that the research was conducted in the absence of any commercial or financial relationships that could be construed as a potential conflict of interest.
