# Supplementary material for: Genome-Wide Association Study Uncovers Novel Genomic Regions Associated With Coleoptile Length in Hard Winter Wheat
Source: Front Genet. 2020 Feb 5;10:1345. doi: 10.3389/fgene.2019.01345 (PMC7025573; doi:10.3389/fgene.2019.01345)

Supplementary Figure S1. Population structure analysis of 298 genotypes of Hard winter wheat association-mapping panel (HWWAMP). The genotype of each line is represented by a colour bar, with four different colours elucidating population structure for K1-K4.

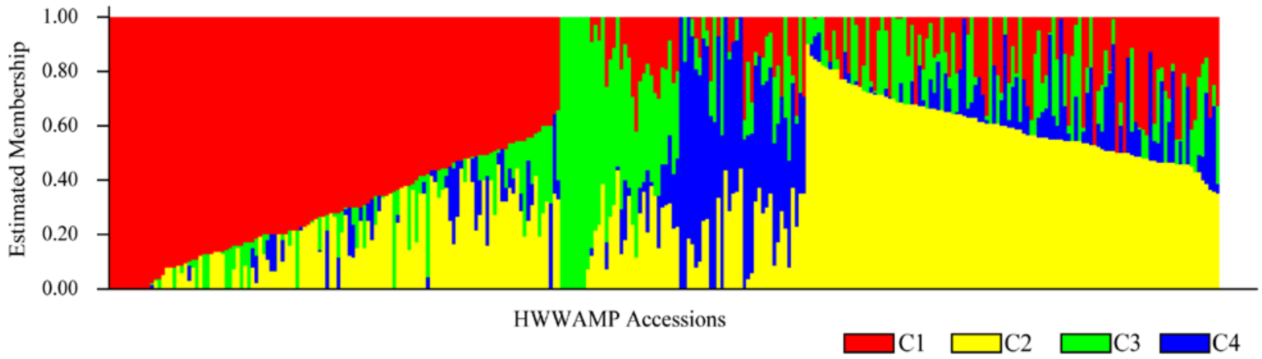

Supplement: Supplementary file 1 [file Image_1.pdf]
