# Supplementary material for: Genome-Wide Association Study Uncovers Novel Genomic Regions Associated With Coleoptile Length in Hard Winter Wheat
Source: Front Genet. 2020 Feb 5;10:1345. doi: 10.3389/fgene.2019.01345 (PMC7025573; doi:10.3389/fgene.2019.01345)
Supplement: Supplementary file 2 [file Table_1.docx]

**Supplementary Table S1.** Average coleoptile length of 298 hard winter wheat association-mapping accessions in three different experiments.

| **Line** | **Coleoptile Length**  **(mm)** | **Line** | **Coleoptile Length**  **(mm)** | **Line** | **Coleoptile Length**  **(mm)** |
| --- | --- | --- | --- | --- | --- |
| A2145 | 72.98 | JUDITH | 68.46 | PRONGHORN | 92.59 |
| A2174-05 | 77.33 | JULES | 73.08 | PROWERS | 93.85 |
| A2180 | 59.79 | KARL_92 | 60.28 | RAWHIDE | 66.62 |
| ABOVE | 66.10 | KAW61 | 105.83 | REDLAND | 62.77 |
| AGATE | 111.08 | KEOTA | 70.26 | RIPPER | 80.46 |
| AKRON | 66.71 | KHARKOF | 91.96 | RITA | 65.47 |
| ALICE | 56.18 | KIOWA | 94.59 | ROBIDOUX | 68.53 |
| ALLIANCE | 60.25 | KIRWIN | 104.05 | RONL | 63.26 |
| ANTELOPE | 75.08 | KS00F5-20-3 | 70.91 | ROSE | 100.00 |
| ANTON | 68.17 | LAKIN | 69.69 | ROSEBUD | 90.07 |
| ARAPAHOE | 74.66 | LAMAR | 93.51 | SAGE | 93.34 |
| ARLIN | 69.15 | LANCER | 88.75 | SANDY | 81.20 |
| AVALANCHE | 67.17 | LARNED | 95.70 | SANTA_FE | 66.85 |
| BAKERS_WHITE | 65.64 | LINDON | 57.60 | SCOUT66 | 102.15 |
| BENNETT | 81.26 | LONGHORN | 107.21 | SD00111-9 | 78.45 |
| BIG_SKY | 80.78 | MACE | 66.81 | SD01058 | 66.33 |
| BILL_BROWN | 62.19 | MCGILL | 64.33 | SD01237 | 80.17 |
| BILLINGS | 83.07 | MILLENNIUM | 81.48 | SD05118 | 72.08 |
| BISON | 91.26 | MIT | 73.42 | SD05210 | 65.53 |
| BOND_CL | 62.36 | MT0495 | 68.61 | SD05W018 | 62.04 |
| BRONZE | 97.06 | MT06103 | 111.09 | SETTLER_CL | 81.63 |
| BUCKSKIN | 92.54 | MT85200 | 65.29 | SHAWNEE | 85.43 |
| BURCHETT | 69.69 | MT9513 | 75.33 | SHOCKER | 70.39 |
| BYRD | 77.00 | MT9904 | 88.85 | SIOUXLAND | 81.53 |
| CAMELOT | 72.10 | MT9982 | 65.60 | SMOKYHILL | 73.38 |
| CAPROCK | 85.90 | MTS0531 | 72.99 | SPARTAN | 79.45 |
| CARSON | 86.77 | NE02558 | 70.14 | STANTON | 78.05 |
| CENTERFIELD | 68.61 | NE04490 | 63.84 | STURDY | 83.89 |
| CENTURA | 86.99 | NE05430 | 57.54 | STURDY_2K | 65.51 |
| CENTURK78 | 96.33 | NE05496 | 76.33 | TAM105 | 70.17 |
| CENTURY | 68.56 | NE05548 | 71.68 | TAM107 | 70.08 |
| CHENEY | 99.21 | NE06545 | 70.28 | TAM107-R7 | 72.34 |
| CHEYENNE | 91.27 | NE06607 | 60.93 | TAM109 | 60.64 |
| CHISHOLM | 70.49 | NE99495 | 68.57 | TAM110 | 81.34 |
| CO03064 | 66.64 | NEKOTA | 78.45 | TAM111 | 81.69 |
| CO03W043 | 77.18 | NELL | 86.44 | TAM112 | 79.41 |
| CO03W054 | 72.32 | NEOSHO | 91.68 | TAM200 | 68.55 |
| CO04025 | 78.14 | NEWTON | 64.67 | TAM202 | 68.32 |
| CO04393 | 65.13 | NI06736 | 74.00 | TAM203 | 66.37 |
| CO04499 | 81.42 | NI06737 | 68.27 | TAM302 | 72.06 |
| CO04W320 | 62.93 | NI07703 | 66.37 | TAM303 | 75.72 |
| CO050337-2 | 72.49 | NI08707 | 68.72 | TAM304 | 70.22 |
| CO07W245 | 71.34 | NI08708 | 66.78 | TAM400 | 76.96 |
| CO940610 | 75.88 | NIOBRARA | 83.38 | TAM401 | 72.84 |
| COLT | 70.09 | NORKAN | 71.77 | TAMW-101 | 62.72 |
| COMANCHE | 98.49 | NORRIS | 87.03 | TANDEM | 78.74 |
| COSSACK | 94.94 | NUFRONTIER | 85.64 | TARKIO | 70.20 |
| COUGAR | 92.78 | NUHORIZON | 89.98 | TASCOSA | 87.54 |
| CREST | 98.59 | NUPLAINS | 59.67 | THUNDER_CL | 76.92 |
| CRIMSON | 101.39 | NUSKY | 61.91 | THUNDERBOLT | 68.05 |
| CULVER | 71.77 | NW03666 | 64.77 | TREGO | 77.31 |
| CUSTER | 65.46 | OGALLALA | 70.54 | TRISON | 72.75 |
| CUTTER | 66.18 | OK_BULLET | 74.59 | TRIUMPH64 | 83.90 |
| DANBY | 71.70 | OK_RISING | 68.74 | TURKEY_NEBSEL | 89.15 |
| DARRELL | 72.51 | OK02405 | 78.82 | TX00V1131 | 78.75 |
| DAWN | 92.34 | OK04111 | 72.06 | TX01A5936 | 71.34 |
| DECADE | 68.55 | OK04415 | 81.52 | TX01M5009-28 | 67.03 |
| DELIVER | 77.22 | OK04505 | 55.26 | TX01V5134RC-3 | 71.06 |
| DENALI | 73.55 | OK04507 | 76.53 | TX02A0252 | 69.20 |
| DODGE | 69.29 | OK04525 | 68.51 | TX03A0148 | 77.07 |
| DUKE | 84.02 | OK05108 | 67.20 | TX03A0563 | 60.78 |
| DUMAS | 64.61 | OK05122 | 60.03 | TX04A001246 | 58.64 |
| DUSTER | 66.27 | OK05134 | 84.41 | TX04M410164 | 84.59 |
| E2041 | 63.89 | OK05204 | 84.83 | TX04M410211 | 80.10 |
| EAGLE | 88.16 | OK05303 | 59.06 | TX04V075080 | 82.34 |
| ENDURANCE | 87.78 | OK05312 | 70.33 | TX05A001188 | 76.03 |
| ENHANCER | 81.14 | OK05511 | 75.47 | TX05A001822 | 71.68 |
| EXPEDITION | 66.92 | OK05526 | 65.70 | TX05V7259 | 68.43 |
| FULLER | 69.34 | OK05711W | 64.09 | TX05V7269 | 83.87 |
| G1878 | 73.49 | OK05723W | 49.70 | TX06A001132 | 68.79 |
| GAGE | 89.95 | OK05830 | 70.79 | TX06A001263 | 63.27 |
| GALLAGHER | 64.07 | OK06114 | 68.01 | TX06A001281 | 63.47 |
| GARRISON | 54.68 | OK06210 | 73.44 | TX06A001386 | 69.63 |
| GENOU | 102.28 | OK06318 | 74.58 | TX06V7266 | 79.79 |
| GENT | 93.66 | OK06319 | 75.56 | TX07A001279 | 69.01 |
| GOODSTREAK | 92.99 | OK06336 | 72.24 | TX07A001318 | 69.59 |
| GUYMON | 76.48 | OK07231 | 77.41 | TX07A001420 | 72.13 |
| HAIL | 59.99 | OK07S117 | 63.56 | TX86A5606 | 92.85 |
| HALLAM | 75.43 | OK08328 | 74.08 | TX86A6880 | 97.98 |
| HALT | 59.88 | OK09634 | 66.50 | TX86A8072 | 76.10 |
| HARDING | 86.46 | OK101 | 62.34 | TX96D1073 | 65.61 |
| HARRY | 71.48 | OK10119 | 77.76 | TX99A0153-1 | 72.34 |
| HATCHER | 68.10 | OK102 | 73.26 | TX99U8618 | 78.13 |
| HEYNE | 65.88 | OK1067071 | 99.61 | VENANGO | 82.12 |
| HG-9 | 97.33 | OK1067274 | 84.86 | VISTA | 67.39 |
| HOMESTEAD | 88.39 | OK1068002 | 71.74 | VONA | 57.70 |
| HONDO | 80.48 | OK1068009 | 90.89 | W04-417 | 65.64 |
| HUME | 91.51 | OK1068026 | 74.15 | WAHOO | 74.81 |
| HV906-865 | 71.94 | OK1068112 | 72.88 | WARRIOR | 74.57 |
| HV9W03-1379R | 67.96 | OK1070267 | 86.31 | WB411W | 67.85 |
| HV9W03-1551WP | 68.01 | OK1070275 | 83.98 | WENDY | 57.35 |
| HV9W03-1596R | 59.44 | ONAGA | 64.19 | WESLEY | 59.67 |
| HV9W05-1280R | 65.44 | OVERLAND | 63.54 | WICHITA | 90.71 |
| HV9W06-504 | 65.40 | OVERLEY | 59.67 | WINDSTAR | 68.71 |
| INFINITY_CL | 74.48 | PARKER | 98.68 | WINOKA | 65.35 |
| INTRADA | 77.99 | PARKER76 | 95.10 | YELLOWSTONE | 63.43 |
| JAGALENE | 69.32 | PETE | 68.47 | YUMA | 60.38 |
| JAGGER | 69.93 | PLATTE | 60.82 | YUMAR | 65.43 |
| JERRY | 74.46 | POSTROCK | 67.06 | - | - |
| JUDEE | 83.19 | PRAIRIE_RED | 80.57 | - | - |
