# Supplementary material for: Genome-Wide Association Study Uncovers Novel Genomic Regions Associated With Coleoptile Length in Hard Winter Wheat
Source: Front Genet. 2020 Feb 5;10:1345. doi: 10.3389/fgene.2019.01345 (PMC7025573; doi:10.3389/fgene.2019.01345)
Supplement: Supplementary file 3 [file Table_2.docx]

**Supplementary Table S2.** Estimation of variance parameters from different sources using linear mixed model (LMM) analyses.

| Variance source | Estimation | Standard error | P value | LL† | UL‡ |
| --- | --- | --- | --- | --- | --- |
| V(Experiment) | 2.16 | 0.19 | 5.25e^-06*^ | 1.48 | 2.84 |
| V(Genotype) | 127.64 | 1.75 | 3.25e^13*^ | 121.45 | 133.84 |
| V(Residuals) | 43.99 | 1.23 | 2.16e^-10*^ | 39.63 | 48.36 |

^*^Significant at α = 0.05. †Lower Limit (2.50%) for Jackknife resampling and ‡Upper Limit (97.50%). Note - replications are nested under the experiment.
