# Supplementary material for: Genome-Wide Association Study Uncovers Novel Genomic Regions Associated With Coleoptile Length in Hard Winter Wheat
Source: Front Genet. 2020 Feb 5;10:1345. doi: 10.3389/fgene.2019.01345 (PMC7025573; doi:10.3389/fgene.2019.01345)
Supplement: Supplementary file 4 [file Table_3.docx]

**Supplementary Table S3**. Distribution of 298 genotypes among four subpopulations based on the ‘Structure’ analysis. The table shows the number of individuals along with average coleoptile length corresponding to each population.

| Pop | No. of Individuals | Average* |
| --- | --- | --- |
| P1 | 120 | 79.13^a^ |
| P2 | 34 | 75.18^ab^ |
| P3 | 33 | 69.91^b^ |
| P4 | 111 | 72.20^b^ |

*different letters are based on LSD and indicate a significant difference at α = 0.05.
