# Supplementary material for: Genome-Wide Association Study Uncovers Novel Genomic Regions Associated With Coleoptile Length in Hard Winter Wheat
Source: Front Genet. 2020 Feb 5;10:1345. doi: 10.3389/fgene.2019.01345 (PMC7025573; doi:10.3389/fgene.2019.01345)
Supplement: Supplementary file 5 [file Table_4.docx]

**Supplementary Table S4.** Summary of significant SNP markers linked to QTLs for coleoptile length detected using genome-wide association analysis of 298 hard winter wheat accessions.

| Marker | Chromosome | Position (cM) | Position (bp) | log10(p-value) | R2 (%) |
| --- | --- | --- | --- | --- | --- |
| GENE-1397_630 | 2A | 9410 | Na | 3.34 | 4.25 |
| IWA423 | 2A | 25023 | Na | 3.35 | 4.25 |
| IWA8513 | 2A | 25970 | Na | 3.35 | 4.25 |
| D_F1BEJMU02JILPD_53 | 2A | 47217 | 15617488 | 3.8 | 5 |
| Kukri_c19434_504 | 2A | 47217 | Na | 3.35 | 4.28 |
| BS00010629_51 | 2A | 47217 | Na | 3.35 | 4.25 |
| BS00011101_51 | 2A | 47217 | Na | 3.35 | 4.25 |
| BS00086364_51 | 2A | 47217 | Na | 3.35 | 4.25 |
| IWA422 | 2A | 47217 | Na | 3.35 | 4.25 |
| RAC875_rep_c88665_100 | 2A | 47217 | 19028876 | 3.35 | 4.25 |
| BS00003867_51 | 2A | 47217 | Na | 3.35 | 4.24 |
| RAC875_rep_c88665_52 | 2A | 47217 | 19028924 | 3.34 | 4.24 |
| BobWhite_c6463_340 | 2A | 47217 | Na | 3.33 | 4.21 |
| tplb0046h22_935 | 2A | 47217 | Na | 3.03 | 3.86 |
| BS00067280_51 | 2B | 16880 | Na | 3.25 | 4.10 |
| D_contig17313_245 | 2D | 2283 | 9344557 | 3.18 | 4.15 |
| Kukri_rep_c75764_261 | 3B | 37028 | Na | 3.64 | 4.68 |
| Tdurum_contig43252_1407 | 3B | 37288 | Na | 3.79 | 5.03 |
| Rht-B1 | 4B | 43090 | 30859246 | 9.69 | 16.69 |
| BS00023766_51 | 4B | 54637 | 31875354 | 3.14 | 3.95 |
| IAAV971 | 4B | 57489 | 40752368 | 7.1 | 10.56 |
| CAP11_c3631_75 | 4B | 59942 | 46621203 | 3.01 | 3.85 |
| BS00040305_51 | 4B | 60391 | 54742322 | 3.15 | 3.95 |
| Excalibur_c56787_95 | 4B | 58095 | 59207875 | 4.4 | 6.03 |
| Excalibur_c17607_542 | 4B | 63000 | 78025799 | 3.73 | 4.89 |
| RAC875_c12495_1391 | 4B | 64038 | 363305994 | 3.64 | 4.74 |
| Kukri_c5502_2513 | 4B | 71461 | 535070561 | 3.14 | 4.03 |
| BobWhite_c665_296 | 4B | 71365 | 539967132 | 3.18 | 3.99 |
| IACX5891 | 4B | 71461 | 543285573 | 3.11 | 3.88 |
| RAC875_rep_c82932_428 | 4B | 115453 | 666049348 | 3.11 | 4.06 |
| RAC875_rep_c82932_407 | 4B | 115453 | 666049369 | 3.14 | 3.93 |
| RAC875_c23027_445 | 4B | 115453 | 666255980 | 3.15 | 3.96 |
| IAAV5175 | 4B | 64257 | Na | 3.9 | 5.15 |
| Kukri_c35140_75 | 4B | 64257 | Na | 3.87 | 5.1 |
| Kukri_c29414_105 | 4B | 64257 | Na | 3.59 | 4.71 |
| IWA7167 | 4B | 71461 | Na | 3.12 | 3.95 |
| Tdurum_contig67535_391 | 5B | 71644 | 536323799 | 4.00 | 5.27 |
| BS00065128_51 | 5B | 110561 | Na | 3.12 | 3.89 |
| RFL_Contig539_1789 | 5B | 112367 | Na | 3.41 | 4.33 |
| BobWhite_c11495_120 | 5B | 112367 | Na | 3.14 | 3.93 |
| IWA1460 | 5B | 116107 | Na | 3.53 | 4.51 |
| Excalibur_c72450_483 | 5B | 182150 | Na | 3.84 | 5.46 |
| IWA3933 | 6B | 64084 | Na | 3.31 | 4.18 |
| BS00065357_51 | 6B | 110447 | 705754074 | 3.31 | 4.19 |
| RAC875_rep_c106238_237 | 6B | 110447 | Na | 3.31 | 4.19 |
| Kukri_rep_c101126_469 | 6B | 110447 | Na | 3.28 | 4.20 |
