# Supplementary material for: Genome-Wide Association Study Uncovers Novel Genomic Regions Associated With Coleoptile Length in Hard Winter Wheat
Source: Front Genet. 2020 Feb 5;10:1345. doi: 10.3389/fgene.2019.01345 (PMC7025573; doi:10.3389/fgene.2019.01345)
Supplement: Supplementary file 6 [file Table_5.docx]

**Supplementary Table S5.** Average coleoptile length of hard winter wheat association mapping panel (HWWAMP) genotypes corresponding to each allele of the most significant marker on the respective chromosome. P-values are based on two-sample t-test assuming unequal variance.

| QTL | SNP | Allele 1  /Allele 2^#^ | Chr. | CL (Avg.) Allele 1 | CL (Avg.) Allele 2 | Difference (mm) |
| --- | --- | --- | --- | --- | --- | --- |
| *QCL.sdsu-2AS* | D_F1BEJMU02JILPD_53 | C/T | 2A | 75.54 | 66.92 | 8.62 |
| *QCL.sdsu-2BS* | BS00067280_51 | T/C | 2B | 76.21 | 72.70 | 3.51 |
| *QCL.sdsu-2DS* | D_contig17313_245 | C/A | 2D | 76.34 | 69.21 | 7.13 |
| *QCL.sdsu-3BS* | Tdurum_contig43252_1407 | T/C | 3B | 80.83 | 72.57 | 8.25 |
| *QCL.sdsu-4BS* | IAAV971 | C/T | 4B | 81.71 | 71.02 | 10.70 |
| *QCL.sdsu-4BL* | RAC875_rep_c82932_407 | A/G | 4B | 76.69 | 70.93 | 5.76 |
| *QCL.sdsu-5BL* | Tdurum_contig67535_391 | C/A | 5B | 85.37 | 74.42 | 10.94 |
| *QCL.sdsu-6BS* | BS00065357_51 | C/T | 6B | 76.64 | 72.08 | 4.56 |
| *Rht-B1* | *Rht-B1* | a/b | 4B | 84.50 | 71.00 | 13.50 |

*Significant at α = 0.05. ^#^Allele 1 is positive allele and Allele 2 is negative allele.
