# Supplementary material for: Genome-Wide Association Study Uncovers Novel Genomic Regions Associated With Coleoptile Length in Hard Winter Wheat
Source: Front Genet. 2020 Feb 5;10:1345. doi: 10.3389/fgene.2019.01345 (PMC7025573; doi:10.3389/fgene.2019.01345)
Supplement: Supplementary file 7 [file Table_6.docx]

**Supplementary Table S6**. Pedigree and accession numbers of the hard winter wheat association mapping panel (HWWAMP) genotypes with coleoptile length longer than 100 mm

| Name | GRIN | Synonym | Program | Pedigree |
| --- | --- | --- | --- | --- |
| CRIMSON | PI 601818 | SD89153 | SDK | TAM105/Winoka |
| SCOUT66 | CItr 13996 | CItr13996 | NEB | Composite of 85 selections from SCOUT, CItr 13546 |
| GENOU | PI 640424 | MTS0031 | MSU | Lew/Tiber//Redwin (MTS92015)/3/Vanguard/Norstar |
| KAW61 | CItr 12871 | KS471238 | KSM | purification and re - release of Kaw Oro//Mediterranean/Hope/3/Early-Blackhull/Tenmarq |
| KIRWIN | CItr 17275 | KS 6623 | KSM | Parker*3/Bison |
| LONGHORN | PI 552813 | PI552813 | APS | NS2630-1/Thunderbird |
| MT06103 |  |  | MSU | Composite cross |
| AGATE | CItr 17463 | NE69442 | NEB | Ponca/3*Cheyenne//Kenya58/Newthatch//2*(Cheyenne/Tenmarq/Mediterranean/Hope)/3/Scout |
