# Supplementary material for: Genome-Wide Association Study Uncovers Novel Genomic Regions Associated With Coleoptile Length in Hard Winter Wheat
Source: Front Genet. 2020 Feb 5;10:1345. doi: 10.3389/fgene.2019.01345 (PMC7025573; doi:10.3389/fgene.2019.01345)
Supplement: Supplementary file 8 [file Table_7.docx]

**Supplementary Table S7**. Chromosome location and nucleotide sequences flanking the significant SNPs associated with coleoptile length.

| Chr. | Marker | Marker sequence** |
| --- | --- | --- |
| 2A | D_F1BEJMU02JILPD_53 | TGGAGAGTCTATATGGAGAGTACCTGCTTAGGATCTGCGAGTCGTTTGCGAG**[T/C]**GCGCCGATGATCCAGTAGATGAGGCTCTGGAAGATGGCGTCCATGAGGCCGTAGCTGAAGTAGAGCAGGAAGGGCCCGGCGTAGCGGCGGCCCTCCTTGAAGTCGATCTTGTCGGGGAATTCGCC |
| 2B | BS00067280_51 | CAGTTGCATGGACGATGGCGATGAAAGTTGACAAGGTACTTGAAAGACTA**[T/C]**CTGGAAGGAAGAAAAAGATGACCGTGGTAAGCCATTCTGAGCTGAAGTGT |
| 2D | D_contig17313_245 | CTGCTCTTAGAATATTCATTGTATGAACTACATTGATTGCTAGATACACACCCATTATAGCAGTGACATATATGATTTGTCAATTTGCATGCAGTGGGTACGTACCGCCTGAATATATCGACGC**[A/C]**GCTATAATCTCTATTAAGTTTGACATATACAGTTTGGGCGTGGTAATCATAAAGTTAATGACAGGACCAATGGGCTACTTTAGAAGTGCTGAAATGTCGCCTGAACAATTCATAGAACTTGTAAG |
| 3B | Tdurum_contig43252_1407 | GCGGTGAGGTCCCCATGGTCACCCAACCTCAGACCATTCTTCAAAGTGTT**[T/C]**GAACTCGTTGACATCGAGGCCAGCGAGGGGATGGCACATGGCGCACACAA |
| 4B | IAAV971 | CATCGGCATTGACGCTCTTCTTCGTATCTTTCCTCTCATTTCCATCCTGGTGCGTACTATGATAACTAGACGACGGGATTTCTGTAGGAAACGGAAGCAC**[T/C]**GTCTTGACTGCATAGCCATTCAAGGGCTCCTCCTTTTCTGGGACCAGATTCTTGTCACTCGAATGGCCTGGTTCACTGACTTCACTTCTCCCTGCGGTGT |
| 4B | RAC875_rep_c82932_407 | AGTAGTGGCCTCAGCTCCAAAGAAGATATGAAAGGAGTCCCGGAACCGAA**[A/G]**TTTACCAGGCAAACGGACACTGAGCTGCCTGTAGCAACGGTGACCACCTC |
| 5B | Tdurum_contig67535_391 | ACGACAGGAGTGGAGCCTCAATCTGTCTCCCCTCGTTGTGTTCGTCATCG**[T/G]**GTCCAGCCGATGCGGGGAGTGCTGCTTCCTCGTCGCCGGCTTTGAATCCC |
| 6B | BS00065357_51 | ACTCTTGTCTAGCGAGGACAACAAACAAGGAATTGAAGGAAAAGGTTTGC**[T/C]**GTGAGGGAGGGCAGTTTCCATCCACTGCTGGGGTGGAATTGATGGTGACG |

**Nucleotide in the parenthesis are SNPs
